# Identification of lipid metabolism related immune markers in atherosclerosis through machine learning and experimental analysis

**DOI:** 10.3389/fimmu.2025.1549150

**Published:** 2025-02-25

**Authors:** Hang Chen, Biao Wu, Kunyu Guan, Liang Chen, Kangjie Chai, Maoji Ying, Dazhi Li, Weicheng Zhao

**Affiliations:** ^1^ Department of Thyroid Breast Vascular Surgery, Banan Hospital of Chongqing Medical University, Chongqing, China; ^2^ Department of Vascular Surgery, Changhai Hospital Affiliated to Naval Medical University, Shanghai, China; ^3^ Cancer Research Centre Nantong, Nantong Tumor Hospital, Nantong, China; ^4^ Pediatrics, Changhai Hospital Affiliated to Naval Medical University, Shanghai, China; ^5^ General Practice, Changhai Hospital Affiliated to Naval Medical University, Shanghai, China; ^6^ Department of Interventional, Shenzhen Traditional Chinese Medicine Hospital, Shenzhen, China

**Keywords:** atherosclerosis, lipid metabolism, machine learning, immune infiltration, biomarkers

## Abstract

**Background:**

Atherosclerosis is a significant contributor to cardiovascular disease, and conventional diagnostic methods frequently fall short in the timely and accurate detection of early-stage atherosclerosis. Abnormal lipid metabolism plays a critical role in the development of atherosclerosis. Consequently, the identification of new diagnostic markers is essential for the precise diagnosis of this condition.

**Method:**

The datasets related to atherosclerosis utilized in this research were obtained from the GEO database (GSE2470, GSE24495, GSE100927 and GSE43292). The ssGSEA technique was first utilized to assess lipid metabolism scores in samples affected by atherosclerosis, thereby aiding in the discovery of important regulatory genes linked to lipid metabolism via WGCNA. Following this, differential expression analysis and functional evaluations were carried out, after which various machine learning approaches were employed to determine significant diagnostic genes for atherosclerosis. A diagnostic model was then developed and validated through several machine learning algorithms. Furthermore, molecular docking studies were conducted to analyze the binding affinity of these key markers with therapeutic agents for atherosclerosis. The ssGSEA technique was also used to measure immune cell scores in atherosclerotic samples, aiding the exploration of the connection between key diagnostic markers and immune cells. Finally, the expression variations of the identified pivotal genes were confirmed through experimental validation.

**Result:**

WGCNA identified 302 lipid metabolism-related genes in atherosclerotic samples, and functional analysis revealed that these genes are associated with multiple immune pathways. Through further differential analysis and screening using machine learning algorithms, APLNR, PCDH12, PODXL, SLC40A1, TM4SF18, and TNFRSF25 were identified as key diagnostic genes for atherosclerosis. The diagnostic model we constructed was confirmed to predict the occurrence of atherosclerosis with high accuracy, and molecular docking studies indicated that these six key diagnostic genes have potential as drug targets. Additionally, the ssGSEA algorithm further validated the association of these diagnostic genes with various immune cells. Finally, the expression levels of these six genes were experimentally confirmed.

**Conclusion:**

Our study introduces novel lipid metabolism-related diagnostic markers for atherosclerosis and emphasizes their potential as immune-related drug targets. This research provides a valuable approach for the predictive diagnosis and targeted therapy of atherosclerosis.

## 1 Introduction

Atherosclerosis stands as a primary contributor to cardiovascular disease, significantly impacting mortality rates and the global disease burden (1, 2). Despite significant advancements in the prevention and treatment of cardiovascular diseases, those affected by atherosclerosis still encounter numerous challenges. Traditional diagnostic methods, including blood lipid testing and imaging techniques, often fail to identify early atherosclerosis in a timely and precise manner. Consequently, many patients are diagnosed only after their condition has progressed to a severe stage, losing critical opportunities for prompt intervention (3). Additionally, most existing treatments primarily focus on reducing cholesterol levels and enhancing hemodynamics, often lacking individualized strategies that target specific pathophysiological mechanisms (4). The advancement of atherosclerosis is closely linked to various factors such as inflammation, lipid metabolism, and immune system responses, complicating the identification of a single biomarker that can adequately capture the disease’s complexity (5–7). Hence, creating innovative diagnostic methodologies and immune markers that can more precisely identify the early phases of atherosclerosis, as well as inform tailored treatment plans, represents a significant goal in modern cardiovascular research.

Atherosclerosis is defined by the buildup of lipids, especially low-density lipoprotein (LDL) cholesterol, in the walls of arteries, leading to the formation of plaques and heightened risk for cardiovascular diseases. Dyslipidemia, characterized by abnormal lipid concentrations in the blood, plays a significant role as a risk factor for both the initiation and advancement of atherosclerosis. The importance of lipid metabolism is fundamental to the pathology of this condition. The liver is crucial for overseeing lipid metabolism, which affects the synthesis, breakdown, and transport of lipoproteins. When lipid metabolism in the liver is disrupted, it can lead to elevated levels of lipids in circulation, particularly LDL cholesterol, which is associated with the formation of atherosclerotic plaques. Research indicates a strong link between disturbances in hepatic lipid metabolism and the development of atherosclerosis, underscoring the liver’s essential function in preserving lipid balance and preventing vascular ailments (8, 9). Atherosclerosis initiation involves the buildup of LDL particles within the arterial intima, where they are subject to changes like oxidation. Oxidized LDL (oxLDL) possesses a notable atherogenic potential, as it stimulates inflammatory reactions and attracts immune cells, such as monocytes and macrophages, to the accumulation site. These immune cells consume oxLDL and convert into foam cells, which represent a key feature of early atherosclerotic lesions. The inflammatory reaction initiated by lipid buildup aggravates endothelial dysfunction, resulting in a detrimental cycle of lipid accumulation and inflammation (10). Additionally, the relationship between lipid metabolism and inflammation plays a vital role in atherosclerosis. Dyslipidemia not only leads to lipid buildup but also triggers an inflammatory response that speeds up plaque formation. For example, elevated triglyceride levels combined with reduced concentrations of high-density lipoprotein (HDL) cholesterol are linked to a greater cardiovascular risk. HDL is recognized for its function in reverse cholesterol transport, a process that helps transfer surplus cholesterol from peripheral tissues to the liver for elimination. An inadequacy in HDL function can disrupt this mechanism, resulting in increased lipid buildup in the arterial walls (11, 12). Recent research has emphasized the significance of specific genes and proteins in lipid metabolism and atherosclerosis development. A notable example is the SORT1 gene, identified as a key regulator of LDL cholesterol levels and the associated risk of atherosclerosis. Changes in this gene can affect lipid metabolism in hepatocytes and macrophages, subsequently impacting the advancement of atherosclerosis (13). Moreover, metabolic routes that include enzymes like HMG-CoA reductase, often inhibited by statins, are essential in the synthesis and regulation of cholesterol. In addition to decreasing LDL cholesterol levels, statins also demonstrate anti-inflammatory properties that aid in their protective function against atherosclerosis (14, 15). In conclusion, the connection between lipid metabolism and atherosclerosis is defined by a multifaceted interaction involving lipid buildup, inflammatory processes, and genetic influences. The presence of dyslipidemia, especially high levels of LDL cholesterol and triglycerides, combined with lower HDL levels, plays a significant role in the progression of atherosclerosis. Grasping these mechanisms is essential for creating effective strategies for the prevention and treatment of cardiovascular diseases.

Machine learning and artificial intelligence are increasingly utilized to identify and predict disease-related biomarkers (16–18). These advanced computational technologies enhance the capacity to analyze complex medical data, resulting in improved diagnostic accuracy and the discovery of novel biomarkers (19–21). For example, a study demonstrated that integrating multiple machine learning algorithms with feature selection methods could enhance the prediction of large-artery atherosclerosis (LAA) by identifying clinical risk factors and metabolites involved in lipid metabolism and aminoacyl-tRNA biosynthesis, achieving an area under the receiver operating characteristic curve (AUC) of 0.92 (22). In another study, researchers used a nonnegative matrix factorization algorithm to classify patients and identify DEGs associated with prognosis, revealing that genes such as IL17C and ACOXL could serve as diagnostic markers for atherosclerosis (23). In our study, we assessed lipid metabolism scores in atherosclerotic samples using the ssGSEA algorithm and investigated the functions of lipid metabolism-related genes within these samples. Subsequently, we employed XGBoost and random forest algorithms to evaluate the scores of lipid metabolism-related genes. Key diagnostic markers for atherosclerosis were identified and experimentally validated. In summary, our study aims to provide new diagnostic markers for patients with atherosclerosis.

## 2 Materials and methods

### 2.1 Samples collection

The datasets examined in this research were obtained from the GEO database, with the downloaded information formatted in MINIML (24). The lipid metabolism score utilizes datasets GSE24702 and GSE24495, where GSE24702 comprises 290 atherosclerotic samples and GSE24495 consists of 113 atherosclerotic samples. For differential and immune analyses, datasets GSE100927 and GSE43292 were employed, encompassing 101 samples from atherosclerotic cases and 62 samples from healthy controls. Genecard employs ‘lipid metabolism’ as a keyword to identify genes with scores exceeding 50, categorizing them as regulatory genes involved in lipid metabolism. The samples are then scored based on these identified genes using the ssGSEA algorithm. In contrast, the immune infiltration score is determined by specific genes associated with 28 distinct types of immune cells, as outlined in the study by Charoentong P et al. (24, 25).

### 2.2 Functional analysis of genes

We accessed the KEGG REST API to gather the most recent KEGG pathway gene annotations for our background dataset. Subsequently, we mapped the genes onto this dataset and applied the R software package clusterProfiler (version 3.14.3) to carry out the enrichment analysis, which ultimately produced gene set enrichment outcomes. For the GO analysis, we utilized the GO annotations for genes sourced from the R package org.Hs.eg.db (version 3.1.0) as our background. The genes were integrated into this background collection through the R package clusterProfiler (version 3.14.3) to execute the enrichment analysis and derive the results of the gene set enrichment (26).

### 2.3 Evaluation and correlation analysis of immune infiltration in atherosclerosis

Utilizing the ssGSEA algorithm from the R package GSVA, an assessment of immune cell infiltration in samples impacted by atherosclerosis was conducted (27, 28). The R package ggstatsplot was employed to generate heat maps associated with immune infiltration. For examining the correlations between quantitative variables not conforming to a normal distribution, Spearman correlation analysis was utilized. Following the classification of essential atherosclerosis-related genes into groups of high and low expression, statistical evaluations were carried out to determine the proportion of each subgroup within their respective categories. The ggplot2 package was used to create stacked bar charts for a visual representation of the statistics.

### 2.4 Molecular docking of key genes with aspirin and atorvastatin

To evaluate the binding affinity between essential genes and medications for atherosclerosis, we conducted an analysis using molecular docking techniques. Our study was significantly enhanced by the use of the CB-Dock2 platform, and we utilized the Vina score to assess the affinity of the genes with the drugs (29). It is widely recognized that a Vina score below -5.0 kcal/mol suggests stronger binding interactions between the two elements.

### 2.5 Constructing diagnostic models

To create a highly accurate and consistently performing diagnostic model for atherosclerosis, we combined several machine learning algorithms in various configurations. The algorithms utilized in this study comprise Elastic Net (Enet), Gradient Boosting Machine (GBM), glmBoost, Least Absolute Shrinkage and Selection Operator (Lasso), Linear Discriminant Analysis (LDA), Naive Bayes, plsRglm, Random Forest (RF), Ridge, Stepglm, Support Vector Machine (SVM), and Extreme Gradient Boosting (XGBoost). For training purposes, the GSE100927 dataset was employed and subsequently validated with the GSE43292 dataset. Each combination of algorithms was assessed based on the Area Under the Curve (AUC) metric, and the one that produced the highest average AUC value was chosen as the best model.

### 2.6 qRT-PCR

Total RNA was initially extracted from atherosclerotic samples utilizing TRIzol reagent, after which qRT-PCR was performed. HiScriptII SuperMix was used for the synthesis of complementary DNA. The qRT-PCR analysis was conducted on an ABI7500 system employing SYBR Green MasterMix. The PCR amplification protocol comprised 45 cycles, beginning with an initial incubation of 10 minutes at 94°C, and then followed by cycles lasting 10 seconds at 94°C and 45 seconds at 60°C. GAPDH served as the internal control in this research. Below is the list of primer pair sequences for the target genes: APLNR (Forward: GCTGTGCCTGTCATGGTGTT, Reverse: CACTGGATCTTGGTGCCATTT), PCDH12 (Forward: CAATGGGAATCCCCCTAAGT, Reverse: TAGGTGGTCCACACTGGTGA), PODXL (Forward: TTTTACTCTTGCCCTCTC, Reverse: CTTTCTTTCTGCCAAGAAAC), SLC40A1 (Forward: CCTACACTCAGGGACTGCGT, Reverse: CCATTATTCCAGTTATAGCT), TM4SF18 (Forward: TCTGGATACTGCCTGGTCATCTCTG, Reverse: AAAAGCATACTCCCAGCCATCAAGG), TNFRSF25 (Forward: GGCCCTTCAAGTGACCCTTG, Reverse: CTCGGTGGAGCAGTCAACAC) and GAPDH (Forward: CGGAGTCAACGGATTTGGTCGTAT, Reverse: AGCCTTCTCCATGGTGGTGAAGAC).

### 2.7 Statistical analyses

The assessment of statistical differences between the two groups was conducted using a T-test. The Spearman method was applied for the correlation analysis. A p value below 0.05 was regarded as statistically significant.

## 3 Results

### 3.1 Developing key regulators of lipid metabolism in atherosclerosis

**Figure 1:**
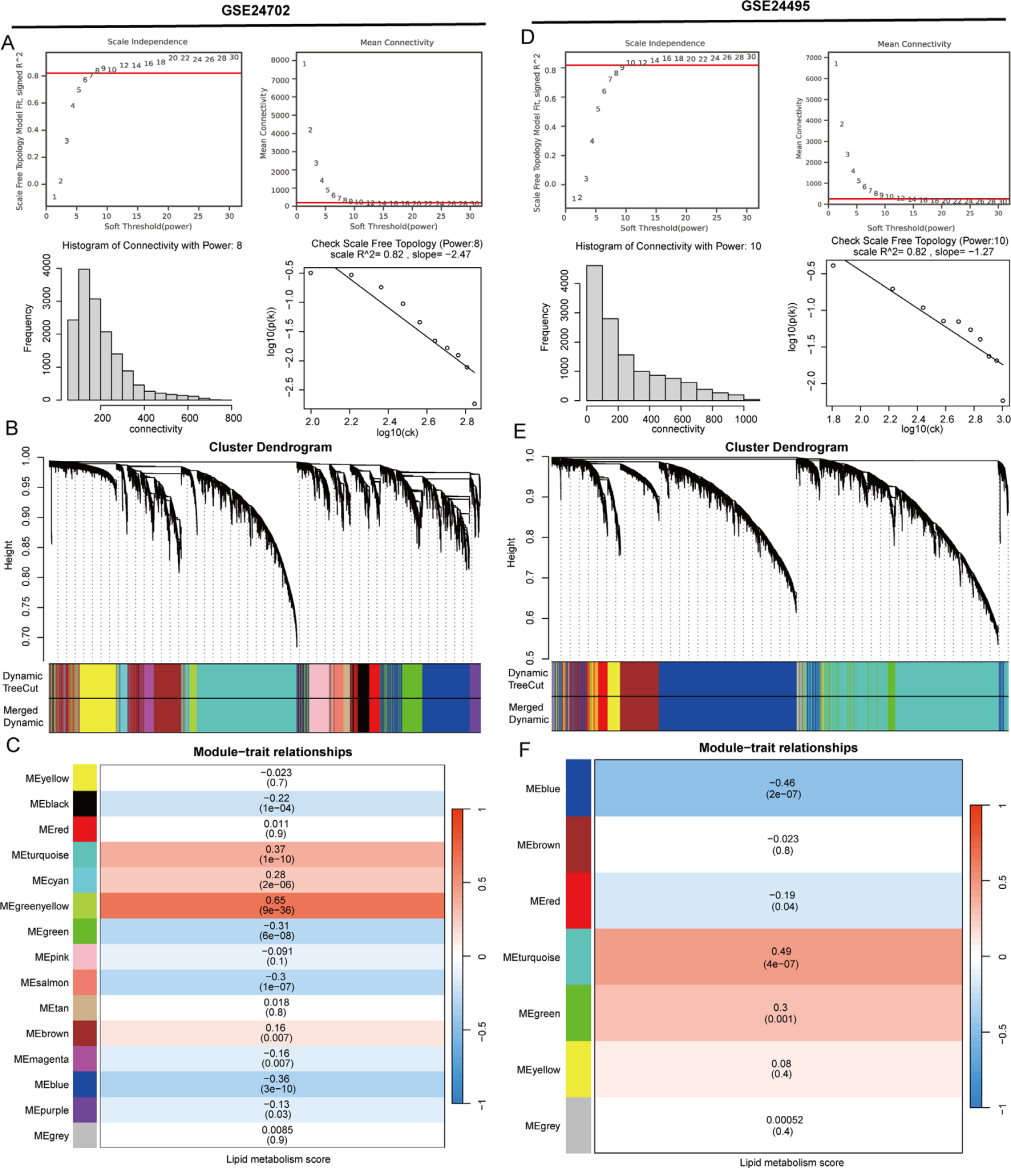
Developing key regulators of lipid metabolism in atherosclerosis. **(A)** The ideal power for soft-thresholding was determined to be 8. **(B)** Construction of a weighted co-expression network was carried out utilizing the chosen power values. **(C)** A heatmap displaying the associations between trait modules was generated. **(D)** The ideal power for soft-thresholding was established at 10. **(E)** A weighted co-expression network was modeled using the selected power values. **(F)** A heatmap illustrating the connections among trait modules was produced.

Utilizing key regulatory genes involved in lipid metabolism sourced from the Genecard database, we applied the ssGSEA algorithm to calculate a lipid metabolism score for each sample from the GSE24702 and GSE24495 datasets. The samples were divided into high and low lipid metabolism groups based on their individual scores. In order to discover potential regulators of lipid metabolism that could play a role in atherosclerosis, we performed a WGCNA analysis using the GSE24702 dataset. To confirm that the network distribution conformed to a scale-free model, we established the weight parameters of the adjacency matrix with a power value of 8 (**Figure 1A**). Following this, we created a weighted co-expression network model that classified all genes into 15 separate modules (**Figure 1B**). By employing the Pearson correlation method, we evaluated the correlation coefficient and p-value between module eigengenes and specific traits, revealing that the green-yellow module showed the highest correlation, with a coefficient of 0.65 (**Figure 1C**). Furthermore, we integrated the GSE24495 dataset into our analysis, using the WGCNA method with a power parameter of 10, which led to the classification of genes into 7 modules. The turquoise module notably exhibited the strongest correlation, achieving a coefficient of 0.49 (**Figures 1D–F**).

### 3.2 Functional analysis of lipid metabolism-related genes

**Figure 2:**
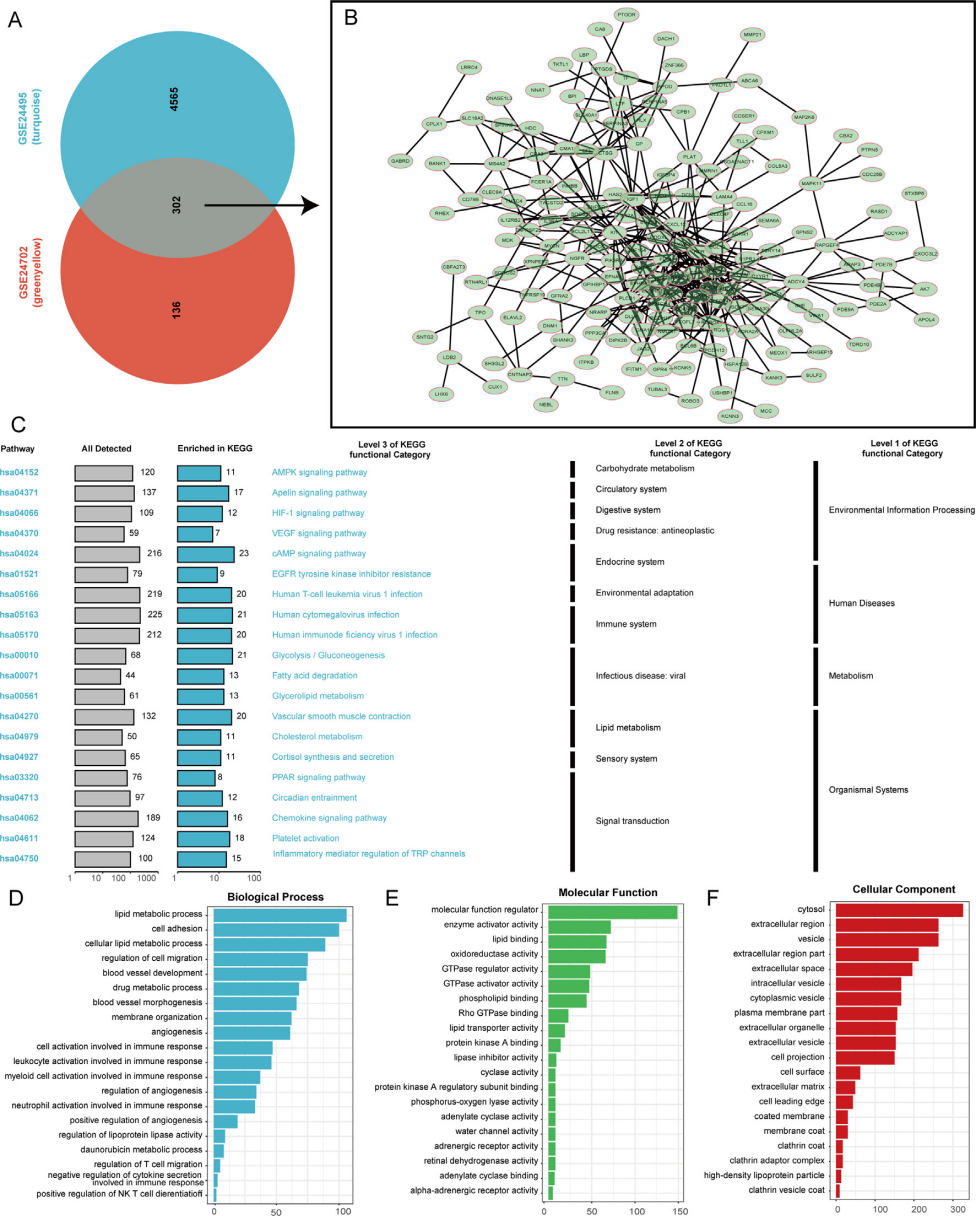
Functional analysis of lipid metabolism-related genes. **(A)** Screening for key lipid metabolism regulatory genes in atherosclerotic samples. **(B)** Interaction network diagram of lipid metabolism regulatory genes. **(C)** KEGG analysis. **(D–F)** GO analysis.

Following the intersection of the two key module genes identified by the WGCNA method in the GSE24702 and GSE24495 data sets, key lipid metabolism genes from 302 atherosclerotic samples were identified. The interactions among these genes were subsequently analyzed using the STRING database (**Figures 2A, B**). The KEGG analysis results reveal that the identified genes are implicated in a variety of biological pathways, underscoring their significance in cellular functions and signaling mechanisms. Specifically, these genes have been associated with several important pathways, including the cAMP signaling pathway, which plays a critical role in transducing extracellular signals into cellular responses. Furthermore, the VEGF signaling pathway is vital for regulating angiogenesis and vascular permeability. The HIF-1 signaling pathway is crucial for cellular responses to hypoxia, while the Apelin and AMPK signaling pathways are involved in energy metabolism and homeostasis. Notably, these genes also exhibit connections to immune-related pathways, which involve the regulation of inflammatory mediators and transient receptor potential (TRP) channels. They are further linked to infections by major viruses, including Human T-cell leukemia virus 1, Human cytomegalovirus, and Human immunodeficiency virus 1 (**Figure 2C**). Moreover, our GO analysis has provided deeper insights into the biological roles of these 302 genes, demonstrating their association with a diverse range of biological processes. These identified processes encompass angiogenesis, which is essential for new blood vessel formation, and lipid metabolism, which is crucial for maintaining energy balance within the body. In addition, the genes are implicated in the regulation of T cell migration, highlighting their potential role in immune responses. Other activities include the regulation of molecular functions, cyclase activity, and specific interactions such as adenylate cyclase binding. The analysis also identifies functions related to cytosolic activities, the development of cellular projections, and the role of high-density lipoprotein particles in lipid transport and cardiovascular health (**Figures 2D–F**).

### 3.3 Machine learning algorithm identifies key genes

**Figure 3:**
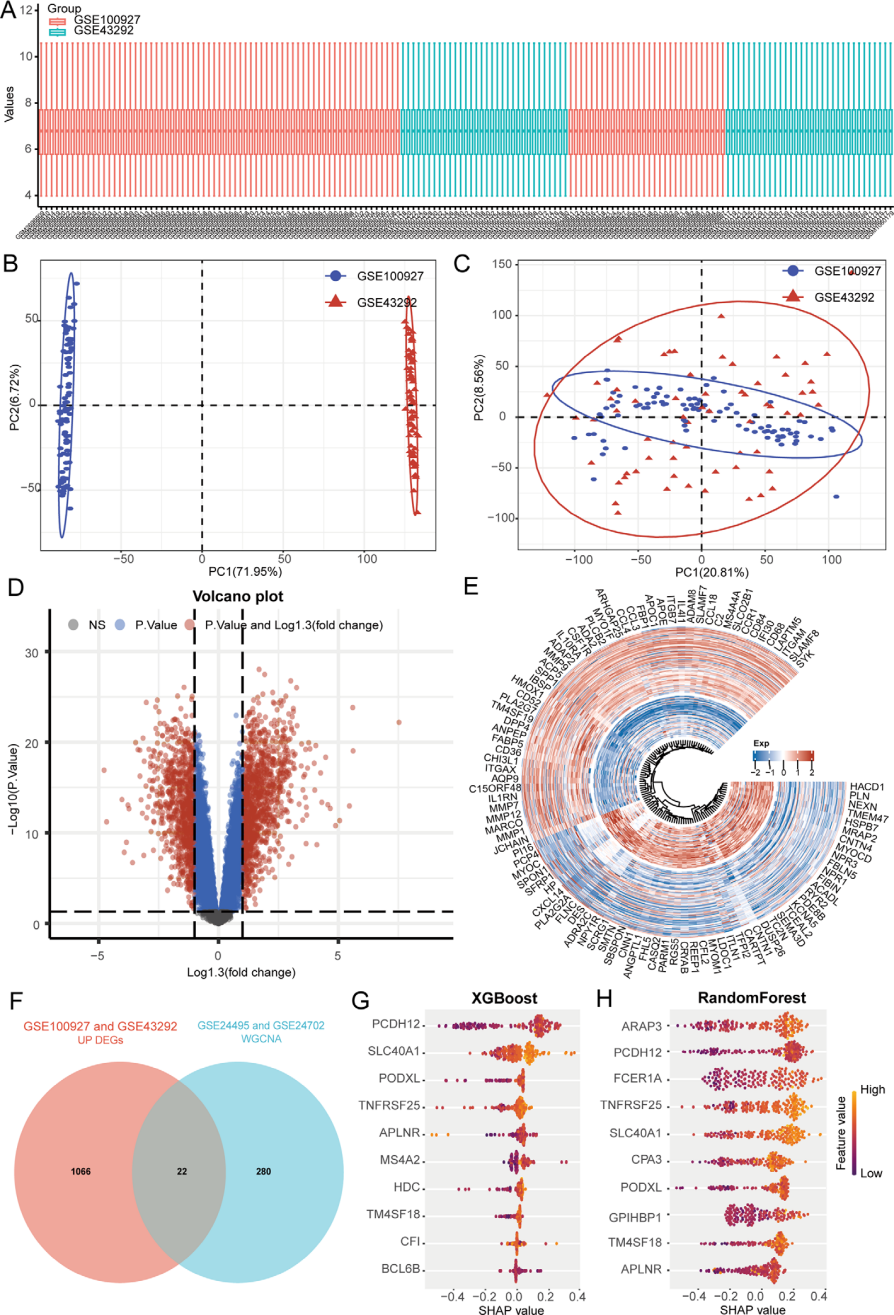
Machine learning algorithm identifies 6 key genes. **(A)** A box plot displays the standardized data. **(B)** PCA results before removing batch effects between different data sets. **(C)** PCA results after removing batch effects between different data sets. **(D, E)** Analysis of differences. **(F)** Genes associated with lipid metabolism in atherosclerosis are identified. **(G, H)** The identification of atherosclerosis-related lipid metabolism genes is achieved using XGBoost and random forest algorithms.

To investigate potential biomarkers related to the progression of atherosclerosis, we acquired two additional datasets connected to atherosclerosis (GSE100927 and GSE43292) from the GEO database, comprising 101 samples from patients with atherosclerosis and 62 samples from healthy controls. Initially, we conducted normalization and integrated the high-throughput sequencing data into one comprehensive dataset for further analysis (**Figure 3A**). Subsequently, we derived PCA results both before (**Figure 3B**) and after batch removal (**Figure 3C**), indicating that the dataset was successfully merged for additional examination. A differential analysis was performed to compare samples from atherosclerotic patients with those from normal controls using the limma package accessible in R. We defined “P <0.05 and log (fold change) > 1.3 or log (fold change) < -1.3” as the hreshold for screening differential mRNA expression. (**Figures 3D, E**). The genes selected for WGCNA analysis displayed a positive correlation with lipid metabolism scores, which are vital in propelling the progression of atherosclerosis. As a result, our differential analysis concentrated solely on genes that are up-regulated in atherosclerotic samples. By utilizing the intersection method, we identified a total of 22 differential genes (**Figure 3F**). Among these, the ten genes most closely associated with atherosclerosis were identified through the utilization of XGBoost and random forest algorithms (**Figures 3G, H**). We determined that APLNR, PCDH12, PODXL, SLC40A1, TM4SF18, and TNFRSF25 are key genes associated with lipid metabolism in samples affected by atherosclerosis.

### 3.4 Expression and localization analysis of atherosclerosis-related genes

**Figure 4:**
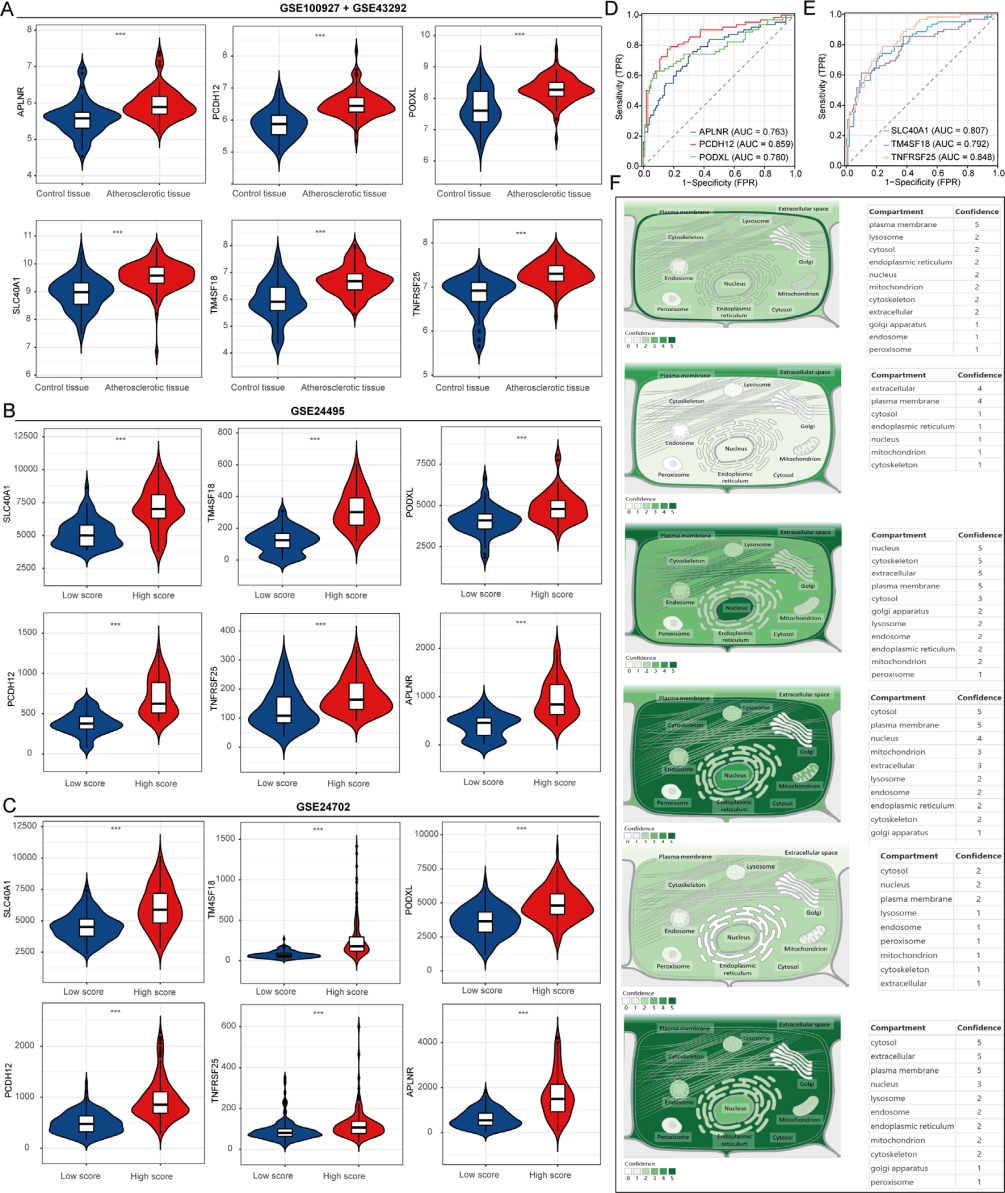
Expression and localization analysis of atherosclerosis-related genes. **(A–C)** Expression analysis of atherosclerosis-related genes. **(D, E)** Predictive value of APLNR, PCDH12, PODXL, SLC40A1, TM4SF18, and TNFRSF25 in the diagnosis of atherosclerosis. **(F)** Localization analysis of APLNR, PCDH12, PODXL, SLC40A1, TM4SF18, and TNFRSF25 in cells. ***P<0.001.

Initially, this study examined changes in the expression of six genes (APLNR, PCDH12, PODXL, SLC40A1, TM4SF18, and TNFRSF25) in atherosclerotic and normal control samples using datasets GSE100927 and GSE43292. The results indicated that APLNR, PCDH12, PODXL, SLC40A1, TM4SF18, and TNFRSF25 exhibited significant upregulation in atherosclerotic samples compared to normal samples (**Figure 4A**). Subsequently, we analyzed the expression differences of these genes in samples with high and low lipid metabolism scores (**Figures 4B, C**). The ROC curve was employed to assess the predictive potential of these key gene markers in atherosclerosis, revealing that the AUC values for APLNR, PCDH12, PODXL, SLC40A1, TM4SF18, and TNFRSF25 were 0.763, 0.859, 0.780, 0.807, 0.792, and 0.848, respectively (**Figures 4D, E**). These findings suggest that these genes may serve as valuable predictors for the diagnosis of atherosclerosis. Finally, the Genecards website was utilized to conduct a subcellular localization analysis of these six genes (**Figure 4F**).

### 3.5 Integration of machine learning algorithms for diagnostic model development

**Figure 5:**
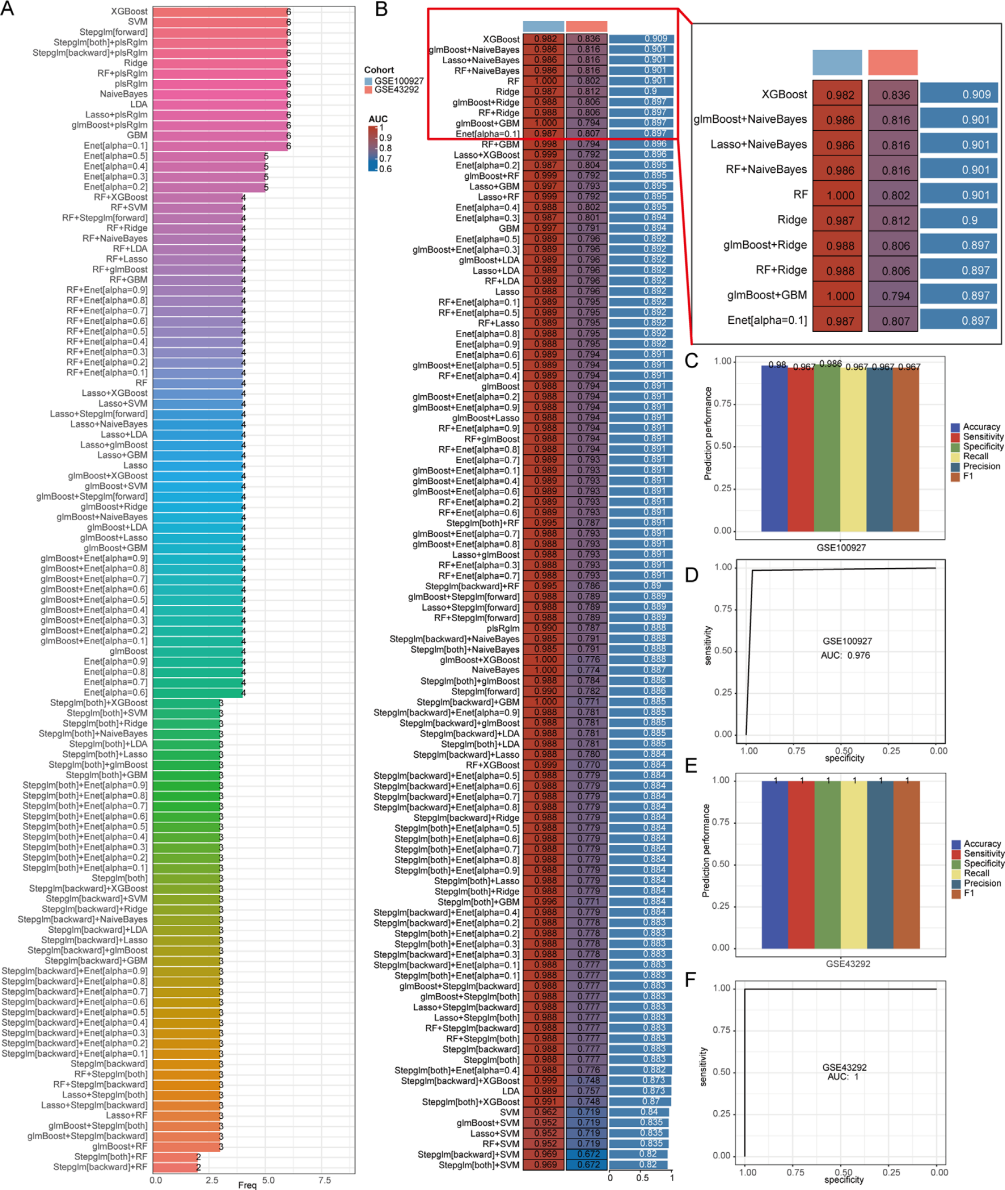
XGBoost algorithm builds diagnostic model. **(A)** Number of genes included in different algorithm combinations. **(B)** AUC values of different algorithm combinations. **(C-F)** Verification of diagnostic model constructed by XGBoost algorithm.

The significance of machine learning in biomedicine is growing, especially when it comes to identifying biomarkers and creating models for diagnosis and treatment. By examining genomic data from patients, machine learning can enhance personalized treatment strategies. In this research, we designed a diagnostic model aimed at atherosclerosis to assist in the early detection of individuals affected by this condition. The model was developed using the GSE100927 dataset and was further validated with the GSE43292 dataset. Out of various algorithm combinations, XGBoost proved to be the most effective for constructing the model. We initially illustrate the count of genes utilized across all algorithm combinations (**Figure 5A**). To make it easier for readers, we have elaborated on the prediction outcomes of the top 10 algorithm combinations (**Figure 5B**). The area under the curve (AUC) for the training data from GSE100927 was recorded at 0.982, while the AUC for the validation dataset GSE43292 stood at 0.836. We then created a diagnostic model of atherosclerosis using the XGBoost algorithm alone, which further supported the findings (**Figures 5C–F**).

### 3.6 Regulatory network of key genes

**Figure 6:**
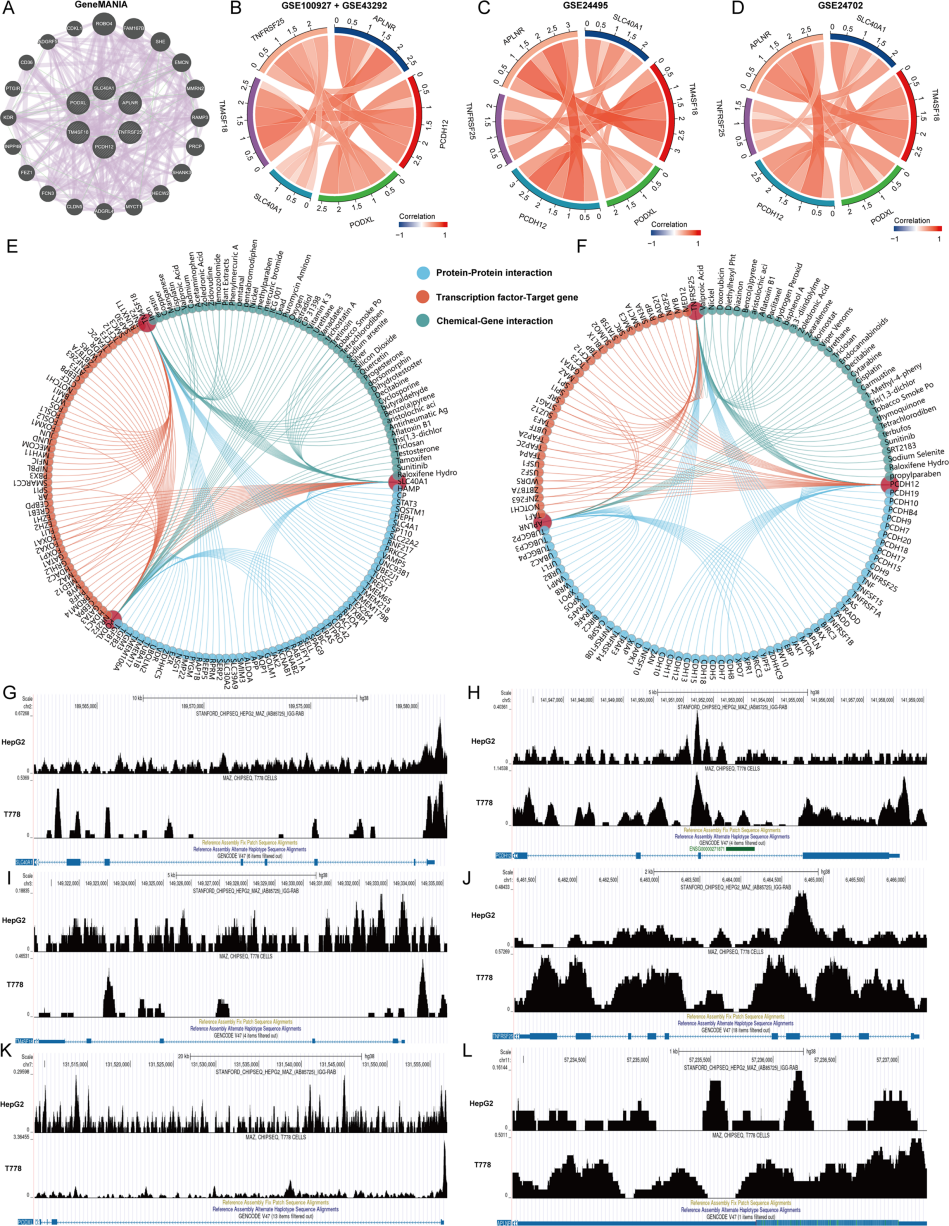
Regulatory network of key genes. **(A)** Interaction network of 6 key genes. **(B–D)** Correlation Chord Diagram of 6 Key Genes. **(E, F)** Analysis of interacting proteins, transcriptional regulators and interacting compounds of 6 key genes. **(G–L)** Transcriptional regulation analysis of 6 key genes and MAZ.

First, we used the GeneMANIA database to analyze the interactions between 6 genes and construct an interaction network (**Figure 6A**). Subsequently, we assessed the correlation of APLNR, PCDH12, PODXL, SLC40A1, TM4SF18, and TNFRSF25 across four datasets: GSE100927, GSE43292, GSE24702, and GSE24495. Our analysis revealed that these six genes consistently exhibited positive correlations within all four datasets (**Figures 5B–D**). Additionally, we utilized the GenDoma database to investigate the interacting proteins of APLNR, PCDH12, PODXL, SLC40A1, TM4SF18, and TNFRSF25, along with transcription factors that have transcriptional regulatory relationships and interacting compounds (**Figures 5E, F**). Notably, we identified the MAZ gene as a common transcriptional regulator for APLNR, PCDH12, PODXL, SLC40A1, TM4SF18, and TNFRSF25. Consequently, we further explored the transcriptional regulatory relationships among these genes using the CistromeDB database (**Figures 5G–L**).

### 3.7 Molecular docking of key atherosclerosis genes and therapeutic drugs

**Figure 7:**
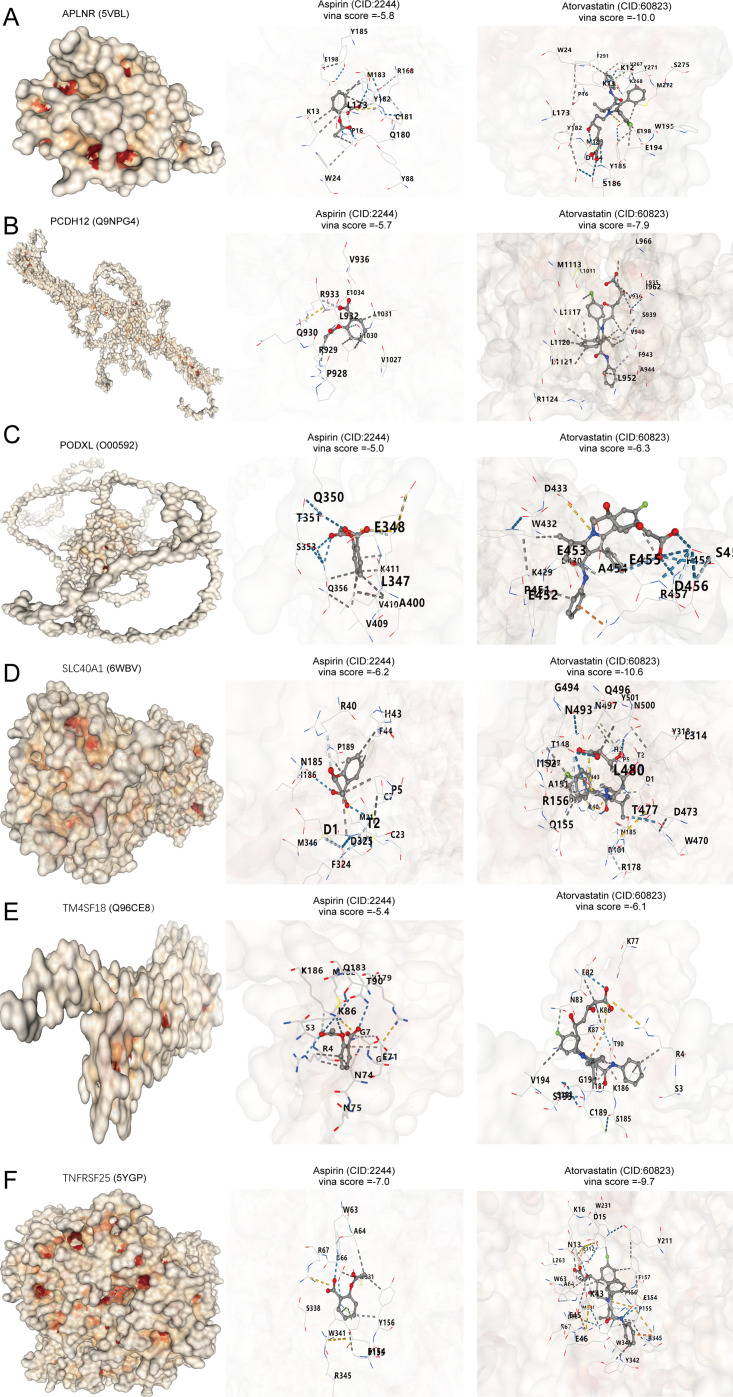
APLNR, PCDH12, PODXL, SLC40A1, TM4SF18, and TNFRSF25 as atherosclerosis drug targets. **(A–F)** Molecular docking of APLNR, PCDH12, PODXL, SLC40A1, TM4SF18, and TNFRSF25 with aspirin and atorvastatin.

Lipid-lowering treatments, particularly statins like atorvastatin, and antiplatelet medications designed to avert thrombosis, with aspirin as a frequently utilized option, are the main therapeutic drugs used for atherosclerosis. In this study, we analyzed the correlation of APLNR, PCDH12, PODXL, SLC40A1, TM4SF18, and TNFRSF25 with atorvastatin and aspirin. Through molecular docking, we discovered that APLNR, PCDH12, PODXL, SLC40A1, TM4SF18, and TNFRSF25 exhibit strong affinities for both atorvastatin and aspirin (**Figures 7A–F**). The Vina docking scores for APLNR with aspirin and atorvastatin were -5.8 and -10, respectively. For PCDH12, the scores were -5.7 and -7.9. PODXL showed scores of -5.0 and -6.3 when docked with aspirin and atorvastatin. SLC40A1 had docking scores of -6.2 and -10.6, while TM4SF18 displayed scores of -5.4 and -6.1. Finally, TNFRSF25 had Vina scores of -7.0 and -9.7 when docked with aspirin and atorvastatin.

### 3.8 Analysis of immune cell infiltration

**Figure 8:**
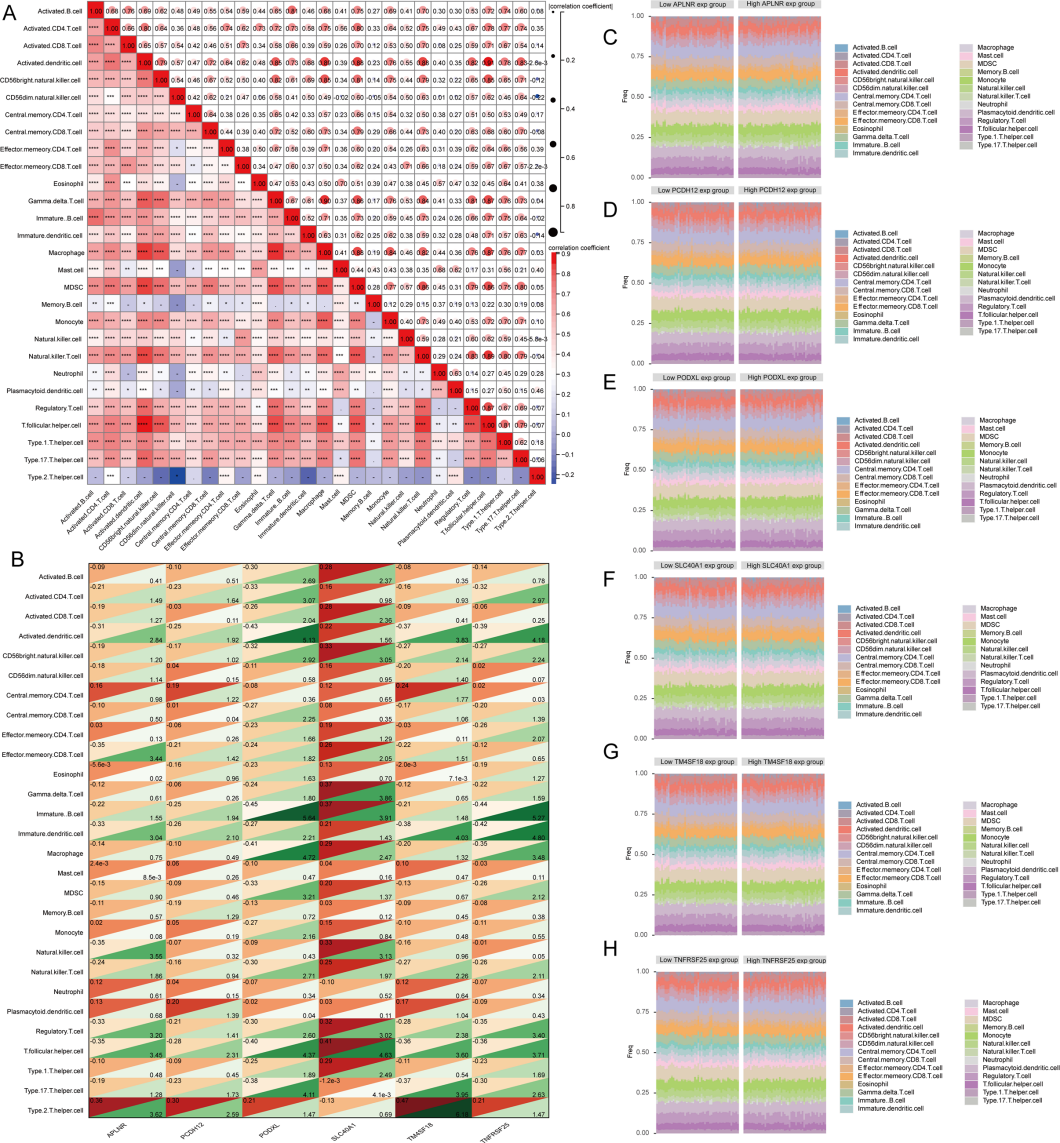
The relationship between key identified genes and immune cells. **(A)** A heatmap depicting the associations among 28 types of immune cells. **(B)** A heatmap displaying the relationships of APLNR, PCDH12, PODXL, SLC40A1, TM4SF18, and TNFRSF25 with the 28 immune cells, where the correlation coefficients are indicated in the upper left corner, and the p-values are presented in the lower right corner. **(C–F)** The percentage of infiltrating immune cells per sample is shown following classification based on the identified genes. ns=P>0.05,*P<0.05, **P<0.01, ***P<0.001.

Recent experimental and clinical evidence suggests that immune mechanisms might contribute to the progression of atherosclerosis. This led us to explore the link between key features of atherosclerosis and the infiltration of immune cells. In their study, Pornpimol et al. identified twenty-eight genes that act as markers for immune cells. We utilized the ssGSEA technique to assess immune cell scores for patients in accordance with this gene set. A heat map was created to illustrate the relationship between the infiltration levels of 28 different types of immune cells. Research findings have demonstrated that the infiltration levels of most immune cells exhibit a positive correlation (**Figure 8A**). We subsequently investigated the interplay among APLNR, PCDH12, PODXL, SLC40A1, TM4SF18, and TNFRSF25 concerning these 28 immune cell types, leading to the creation of an additional heat map (**Figure 8B**). The results indicate that the expression of APLNR is notably associated with the infiltration levels of mast cells and eosinophils, in contrast to PCDH12 expression, which is primarily related to central memory CD8 T cell infiltration levels. Furthermore, PODXL expression exhibited a significant correlation with the infiltration of plasmacytoid dendritic cells. Additionally, SLC40A1 expression showed a strong correlation with the infiltration levels of type 17 T helper cells, while TM4SF18 expression was significantly associated with the infiltration of eosinophils. Lastly, TNFRSF25 expression exhibited a substantial correlation with central memory CD4 T cell infiltration levels. Atherosclerotic samples were organized based on the expression levels of APLNR, PCDH12, PODXL, SLC40A1, TM4SF18, and TNFRSF25. A heat map was used to visually represent the abundance of immune cells within each sample, categorizing them into high and low expression levels, and employing distinct colors to represent various immune cell types (**Figures 8C–H**).

### 3.9 Expression verification of key genes for atherosclerosis

**Figure 9:**
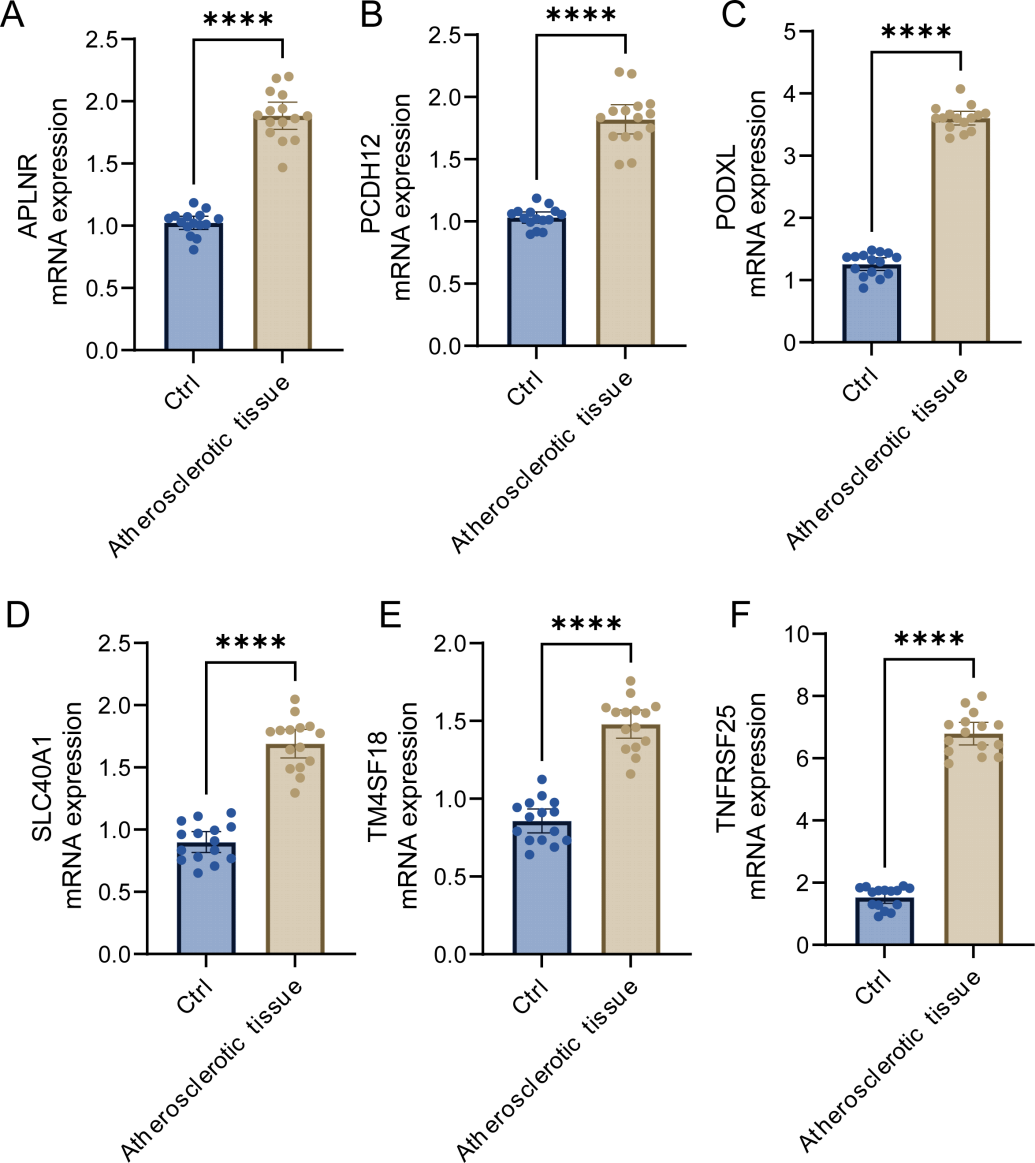
Expression analysis of key genes. **(A–F)** Expression analysis of APLNR, PCDH12, PODXL, SLC40A1, TM4SF18, and TNFRSF25 in atherosclerotic samples and normal samples. ****P<0.0001.

Through our investigation, we discerned the crucial functions of APLNR, PCDH12, PODXL, SLC40A1, TM4SF18, and TNFRSF25 within the framework of atherosclerosis. To facilitate further research, we conducted qRT-PCR detection on sera from five atherosclerosis patients and five control samples. Our findings indicated that the expression levels of APLNR, PCDH12, PODXL, SLC40A1, TM4SF18, and TNFRSF25 were significantly elevated in atherosclerotic samples compared to those found in normal control samples (**Figures 9A–F**). This evidence corroborates our earlier analysis and implies that these markers could function as promising diagnostic tools for atherosclerosis.

## 4 Discussion

The combination of multi-omics analysis and artificial intelligence is propelling the development and application of disease markers in a more precise and personalized direction. Despite facing numerous challenges, its potential is undoubtedly significant (30–33). Recent studies focused on discovering biomarkers for atherosclerosis are advancing rapidly, emphasizing both the molecular mechanisms that drive the disease and the development of innovative diagnostic tools. Atherosclerosis, a chronic inflammatory disorder, is characterized by the accumulation of plaques in the arterial walls and is a major contributor to cardiovascular diseases, often remaining asymptomatic until advanced stages. This highlights the critical importance of early detection and continuous monitoring through reliable biomarkers.

Lipid metabolism comprises a series of biochemical processes that involve the synthesis, breakdown, and transport of lipids, which are essential for maintaining cellular function and energy balance. The primary processes of lipid metabolism include cholesterol synthesis, triglyceride metabolism, lipoprotein transport, and fatty acid oxidation. Each of these processes significantly contributes to the development of atherosclerosis, a chronic inflammatory disease characterized by the accumulation of lipids in the arterial walls. Cholesterol is primarily synthesized in the liver and is essential for maintaining cell membrane integrity, hormone production, and bile acid formation. The regulation of cholesterol levels is crucial, as excessive cholesterol can lead to its deposition in arterial walls, resulting in the formation of atherosclerotic plaques. Dysregulation of cholesterol metabolism, particularly through increased synthesis or decreased clearance, is closely linked to the development of atherosclerosis. For instance, high levels of low-density lipoprotein cholesterol (LDL-C) are a well-established risk factor for atherosclerosis, as LDL particles can penetrate the endothelium and become oxidized, triggering inflammatory responses that contribute to plaque formation. Triglycerides serve as the primary form of stored energy in the body and are transported in the bloodstream by lipoproteins. Elevated triglyceride levels are associated with an increased risk of atherosclerosis. When triglycerides are broken down, free fatty acids are released, which can be taken up by various tissues for energy. However, excessive accumulation of triglycerides can lead to lipotoxicity, promoting inflammation and endothelial dysfunction, both of which are critical in the pathogenesis of atherosclerosis (34, 35). In summary, the major processes of lipid metabolism—cholesterol synthesis, triglyceride metabolism, lipoprotein transport, and fatty acid oxidation—are intricately linked to the development of atherosclerosis. Dysregulation of these processes can lead to lipid accumulation, inflammation, and oxidative stress, all of which contribute to the pathogenesis of atherosclerosis. Understanding these mechanisms is crucial for developing targeted therapies aimed at preventing and treating this prevalent cardiovascular disease. In our study, we identified APLNR, PCDH12, PODXL, SLC40A1, TM4SF18, and TNFRSF25 as the most relevant genes associated with lipid metabolism and atherosclerosis, highlighting their potential as diagnostic and immune markers. Machine learning algorithms have emerged as powerful tools for developing diagnostic models across various medical fields. Their strength lies in the ability to analyze large datasets, identify complex patterns, and enhance diagnostic accuracy. All machine learning techniques incorporate feature selection methods that help pinpoint the variables most relevant to diagnosis. This approach not only improves model performance but also reduces data complexity, facilitating easier interpretation. Utilizing multiple machine learning algorithms, we developed a diagnostic model related to atherosclerosis, achieving an average AUC of 0.909, which demonstrates the model’s exceptional diagnostic capability.

Atherosclerosis is progressively recognized as a long-lasting inflammatory disorder characterized by the infiltration of various immune cell types into the walls of arteries, a process that is essential for the progression of the disease. The immune microenvironment found in atherosclerotic plaques is intricate, comprising various immune cell categories that play roles in both the onset and progression of the illness. Evidence indicates that the infiltration of immune cells significantly contributes to atherosclerosis. For example, research has pinpointed multiple immune cells, such as macrophages, T cells, and dendritic cells, that invade atherosclerotic lesions. These cells may display either pro-inflammatory or anti-inflammatory characteristics, thereby affecting the plaques’ overall inflammatory status. Significantly, activated T lymphocytes, particularly the CD4+ and CD8+ T cell populations, have been associated with the instability and rupture of plaques, which are critical contributors to acute cardiovascular events such as myocardial infarction and stroke (36, 37). Immune cells have the capacity to release pro-inflammatory cytokines, including IL-1β and TNF-α, thereby attracting more immune cells to the inflammatory site and facilitating the development of atherosclerotic plaques (38). The findings of our study suggested a notable relationship between APLNR expression and the levels of mast cell and eosinophil infiltration. Conversely, PCDH12 expression was mainly linked to the infiltration levels of central memory CD8 T cells. Moreover, a noteworthy correlation was found between the expression of PODXL and the infiltration levels of plasmacytoid dendritic cells. In addition, SLC40A1 expression revealed a significant association with the infiltration levels of type 17 T helper cells, whereas TM4SF18 expression displayed a considerable correlation with the levels of eosinophil infiltration. In summary, the relationship between immune infiltration and atherosclerosis is complex and involves dynamic interactions among various immune cell types that can either promote or inhibit the progression of atherosclerosis. Our findings are essential for the development of effective immunotherapies designed to mitigate the inflammatory processes underlying atherosclerosis and enhance cardiovascular outcomes.

While our study presents several important findings, the dataset primarily derived from the GEO database may not fully encapsulate the characteristics of all populations. This limitation affects the generalizability of our results, highlighting the necessity for future research to validate our findings in larger and more diverse samples. Although we identified potential biomarkers, their specific biological functions in atherosclerosis were not thoroughly investigated through animal or clinical experiments. This gap may hinder our understanding of their potential as therapeutic targets. In conclusion, although this study offers new insights into the early diagnosis of atherosclerosis, these limitations must be addressed in future research to achieve a more comprehensive understanding of the relationship between lipid metabolism and atherosclerosis.

## 5 Conclusion

Our study employed various machine learning algorithms to identify that APLNR, PCDH12, PODXL, SLC40A1, TM4SF18, and TNFRSF25, among the lipid metabolism genes, can serve as diagnostic markers in patients with atherosclerosis and are associated with atherosclerotic immune infiltration.

## Data Availability

The original contributions presented in the study are included in the article/supplementary material. Further inquiries can be directed to the corresponding authors.
